# Adaptation of the Oxygen Sensing System during Lung Development

**DOI:** 10.1155/2022/9714669

**Published:** 2022-02-18

**Authors:** Karin M. Kirschner, Simon Kelterborn, Herrmann Stehr, Johanna L. T. Penzlin, Charlotte L. J. Jacobi, Stefanie Endesfelder, Miriam Sieg, Jochen Kruppa, Christof Dame, Lina K. Sciesielski

**Affiliations:** ^1^Institute of Translational Physiology, Charité-Universitätsmedizin Berlin, Corporate Member of Freie Universität Berlin and Humboldt-Universität zu Berlin, 10117 Berlin, Germany; ^2^Department of Neonatology, Charité-Universitätsmedizin Berlin, Corporate Member of Freie Universität Berlin and Humboldt-Universität zu Berlin, 10117 Berlin, Germany; ^3^Institute of Biometry and Clinical Epidemiology, Charité-Universitätsmedizin Berlin, Corporate Member of Freie Universität Berlin and Humboldt-Universität zu Berlin, 10117 Berlin, Germany; ^4^Institute of Medical Informatics, Charité-Universitätsmedizin Berlin, Corporate Member of Freie Universität Berlin and Humboldt-Universität zu Berlin, 10117 Berlin, Germany

## Abstract

During gestation, the most drastic change in oxygen supply occurs with the onset of ventilation after birth. As the too early exposure of premature infants to high arterial oxygen pressure leads to characteristic diseases, we studied the adaptation of the oxygen sensing system and its targets, the hypoxia-inducible factor- (HIF-) regulated genes (HRGs) in the developing lung. We draw a detailed picture of the oxygen sensing system by integrating information from qPCR, immunoblotting, *in situ* hybridization, and single-cell RNA sequencing data in *ex vivo* and *in vivo* models. HIF1*α* protein was completely destabilized with the onset of pulmonary ventilation, but did not coincide with expression changes in *bona fide* HRGs. We observed a modified composition of the HIF-PHD system from intrauterine to neonatal phases: *Phd3* was significantly decreased, while *Hif2a* showed a strong increase and the *Hif3a* isoform *Ipas* exclusively peaked at P0. Colocalization studies point to the *Hif1a-Phd1* axis as the main regulator of the HIF-PHD system in mouse lung development, complemented by the *Hif3a-Phd3* axis during gestation. *Hif3a* isoform expression showed a stepwise adaptation during the periods of saccular and alveolar differentiation. With a strong hypoxic stimulus, lung *ex vivo* organ cultures displayed a functioning HIF system at every developmental stage. Approaches with systemic hypoxia or roxadustat treatment revealed only a limited *in vivo* response of HRGs. Understanding the interplay of the oxygen sensing system components during the transition from saccular to alveolar phases of lung development might help to counteract prematurity-associated diseases like bronchopulmonary dysplasia.

## 1. Introduction

An adequate oxygen homeostasis is essential for proper development and life. During intrauterine gestation, transplacental oxygenation is precisely regulated by complex molecular mechanisms that control oxygen sensing and expression of downstream target genes. This system (i) requires continuous adaptation due to the logarithmic growth of the fetal body and (ii) experiences a dramatic change at birth when the low oxygen partial pressure (pO_2_) rapidly increases to the high levels of air-breathing life. Low oxygen tension during embryonic and fetal development is essential for proper vascular development and organogenesis. This has become evident for lung morphogenesis [1] and other organ developmental programs [2]. Premature birth, however, causes an unexpected, fundamental disturbance of the overall developmental program as the excessive postnatal increase in oxygen tension downregulates the expression of HIF-regulated genes (HRGs). In very preterm infants, this premature switch is thought to cause diseases like bronchopulmonary dysplasia, retinopathy, and anemia of prematurity as they result from an abrupt downregulation of HRGs that subsequently respond by an overwhelming expression [3–5].

One of the main cellular responses to a lower pO_2_ or hypoxic-ischemic conditions is the stabilization of the transcription factor hypoxia-inducible factor (HIF). HIF is a heterodimer characterized by a constitutive *β*-subunit (HIF1*β*) and an oxygen-dependently regulated *α*-subunit (HIF1*α*, HIF2*α*, or HIF3*α*). It transcriptionally induces hundreds of genes which modulate oxygen availability, cell metabolism, and cell growth [6]. In normoxia, HIF*α* proteins are hydroxylated by prolyl-4-hydroxylases (PHD1, PHD2, or PHD3). Hydroxylation of HIF*α* allows binding of the von Hippel-Lindau (VHL) tumor suppressor protein and subsequent degradation of the HIF*α* subunit by the proteasome. The HIF hydroxylases are considered the cellular oxygen sensors as their activity on HIF requires oxygen as cofactor [7]. This cellular oxygen sensing mechanism is essential for proper development as the *Phd2* and *Vhl* as well as the *Hif1a* or *Hif2a* knockout mice die *in utero* or shortly after birth [8–10]. In the lung, HIF2*α* is necessary for normal alveolar development and surfactant production [11, 12]. In contrast, the *Hif3a+Nepas* knockout is viable but shows abnormal heart development and lung remodeling [13]. HIF3*α* has contrasting enhancer and repressor functions. In mouse, there are three isoforms (10 in humans): The full-length transcripts *Hif3a* and *Nepas* (*neonatal and embryonic PAS protein*) and the truncated transcript *Ipas* (*inhibitory Pas domain protein*). In comparison to HIF1*α* or HIF2*α*, HIF3*α* and NEPAS lack the C-terminal transactivation domain (C-TAD) and their oxygen-dependent degradation domain (ODDD) only contains one proline hydroxylation site, which is efficiently hydroxylated by all three PHDs. HIF3*α* and NEPAS are both able to dimerize with HIF1*β* and thereby suppress HIF1 or HIF2 activity [13, 14]. In addition to this inhibitory function, they are also necessary for full hypoxic induction of some HRGs but with far weaker transcriptional stimulation than HIF1/2*α* as they lack the C-TAD. The shortest isoform IPAS lacks not only the C-TAD but also the entire ODDD, making it independent from PHD-mediated degradation. It acts as a dominant negative regulator of HIF1 by forming an inactive complex with HIF1*α* - and the human orthologue HIF3*α*-4 also with HIF2*α* [15, 16].

The interplay of the HIF*α* proteins and the PHD enzymes determines whether hypoxic signaling is established upon a challenge or not. In acute hypoxia, the PHDs are inhibited and HIF*α* is stabilized leading to HRG transcription. In chronic hypoxia, however, the PHD system is reactivated after an adaptation period to prevent the cells from necrosis due to prolonged HIF signaling [17]. It is unclear whether gestation should be considered such a chronic hypoxic state since it is ontogenetically necessary and thereby rather to be considered a “physiological hypoxia”. At premature birth, the physiological oxygen homeostasis is significantly perturbed as there is too much oxygen too early. The influence of hyperoxia on HRGs is only poorly investigated due to its minor clinical relevance in adults. In very premature neonates, however, it is highly relevant because this relative hyperoxia suppresses HRGs such as *VEGFA* and *EPO* [18] and thereby causes disorders of neonatal adaptation. Under the hypothesis that the oxygen sensing system of the lung continuously adapts to the exponential growth of the fetus and to the change in intra- vs. extrauterine pO_2_, the spatiotemporal expression pattern of the oxygen sensors and effectors regulating expression of *bona fide* HRGs was investigated. The understanding of this developmental process could contribute to strategies for prevention of impaired vascularization in bronchopulmonary dysplasia in extremely premature infants.

## 2. Materials and Methods

### 2.1. Animals

C57BL/6J mice from different developmental stages and sexes were used. The developmental stage was determined using Theiler stages as provided by the eMouse Atlas (http://www.emouseatlas.org/emap/home.html, after [19]). All animal procedures were approved by the local animal welfare authorities (LaGeSo Berlin, Germany: T0018/17, T0046/20, T0063/20, and T-CH0019/20).

### 2.2. Animal Experiments

Adult female C57BL/6J mice were treated *p.o.* with vehicle or 600 mg/kg b.w. (body weight) roxadustat (FG-4592, Cayman, #15294), dissolved in 5% DMSO mixed into nut nougat cream, for 8 h. All animals were housed in cages under environment-controlled conditions with a constant 12 h/12 h light/dark cycle, ambient temperature, 40-60% relative humidity, and access to food and water *ad libitum*. All animal experimental procedures were approved by the local animal welfare authorities (LaGeSo: G0133/18) and followed institutional as well as ARRIVE guidelines.

### 2.3. Tissue Preparation

Specimens were prepared from the snap-frozen lungs of adult female BALB/C mice (10-12 wks) and adult male Wistar rats (10-12 wks) exposed to room air or 8% oxygen for 6 h (rats) or 8 h (mice), respectively. The generation of the samples has been described elsewhere [20]. The lungs from vehicle- or roxadustat-treated mice were equally handled. Mouse pups for RNAscope® analysis were anaesthetized with an *i.p.* injection of ketamine (100 mg/kg), xylazine (20 mg/kg), and acepromazine (3 mg/kg) and then transcardially perfused as described previously [3]. The lungs were perfusion-fixed with PBS (pH 7.4), followed by perfusion with 4% paraformaldehyde (Sigma-Aldrich, #158127, pH 7.4). The lungs were postfixed at 4°C for 1 day, embedded in paraffin (Sigma-Aldrich, #76242), and processed for histological staining.

### 2.4. Lung *Ex Vivo* Organ Culture

The lungs were dissected at the indicated ages. Specimens of a maximal diameter of 500 *μ*m were cultured on transwell filters (Corning #3450) at the liquid-air interface in DMEM/Ham's F12 medium (Bio&Sell, #BS.FG4815) containing 10% FBS (Sigma, F7524) and 1% penicillin/streptomycin (Bio&Sell, #BS.A2212) for 21 h at 37°C at the indicated % O_2_ and 5% CO_2_ in humidified conditions.

### 2.5. RNA Extraction and Quantitative PCR

Total RNA was extracted as described [21]. 1000 ng total RNA was reverse-transcribed with SuperScript™ III reverse transcriptase (Thermo Fisher, #18080085) and random hexamers (Thermo Fisher, #SO142) according to the manufacturer's instructions. Quantitative PCRs were run on a StepOnePlus cycler (Life Technologies) with in-house designed and validated intron-spanning primers or probe-based assays (Supplementary Table S1). Absolute mRNA quantification was achieved by comparison with a standard curve from serial dilutions of PCR template (gBlocks from Integrated DNA Technologies, USA). Expression values below 32 mRNA molecules per 10^6^*β-actin* mRNA molecules were considered physiologically irrelevant (depicted by a dashed line in the respective plots).

### 2.6. Protein Extraction and Immunoblotting

Protein extraction and immunoblotting of HIF1*α* protein were performed as described [22], using anti-HIF1*α* antibody (Novus, #NB100-479) and anti-*β-actin* antibody (Sigma-Aldrich, #Mab1501R).

### 2.7. Single-Cell (sc)RNA-seq Analysis

scRNA-seq data from the preprint [23] were downloaded from the NCBI GEO database (Series GSE165063 and GSE160876). Nonviable, immune, and red blood cells were not included in the original lung scRNA-seq dataset. The allocation of the single cells to cellular subcategories was accomplished by marker gene expression as described in [23]. One replicate, each consisting of 4 pooled mouse lung preparations, from the time points E15, E18, P0, P3, P7, P14, and adult (P64) was selected by the highest transcript/cell ratio for further analysis. Analysis was performed as described [23] using SCTransform [24] for normalization of each individual time point and Seurat 4.0.5 [25] for integration of the datasets.

### 2.8. RNAscope *In Situ* Hybridization

RNAscope® assay was performed according to the manufacturer's protocol (ACD, Technical note 323110). 1.5 *μ*m sections of the formalin-fixed, paraffin-embedded lungs were stained with a C1-probe against *Hif1a*, *Hif2a*, or *Hif3a* (ACD, #313821, 314371, 810691) or a C3-probe against *Phd1*, *Phd2*, or *Phd3* (ACD, #414311-C3, 315491-C3, 434931-C3) in combination with the Opal 650 reagent pack (Akoya Biosciences, #FP1496001KT). DAPI was used as counterstaining. Sections were imaged with an Eclipse Ti2 imaging system (Nikon).

### 2.9. Statistics

Data were analyzed using GraphPad Prism 9 and are presented as dot plots with the median or as bar charts with individual dots. For change point detection in the developmental data sets, a newly validated algorithm was applied which controls for the confounder PCR variation [26]. Point estimates and confidence intervals can be found in Supplementary Table S2. Change points were considered relevant if they displayed a change of at least ±factor 2. For all other analyses, nonparametric Mann-Whitney *U* tests were performed. *p* values were not adjusted for multiple testing.

## 3. Results

### 3.1. HIF1*α* Protein Is Completely Destabilized with the Onset of Lung Ventilation but without Effect on the Expression of *Bona Fide* HRGs

**Figure 1:**
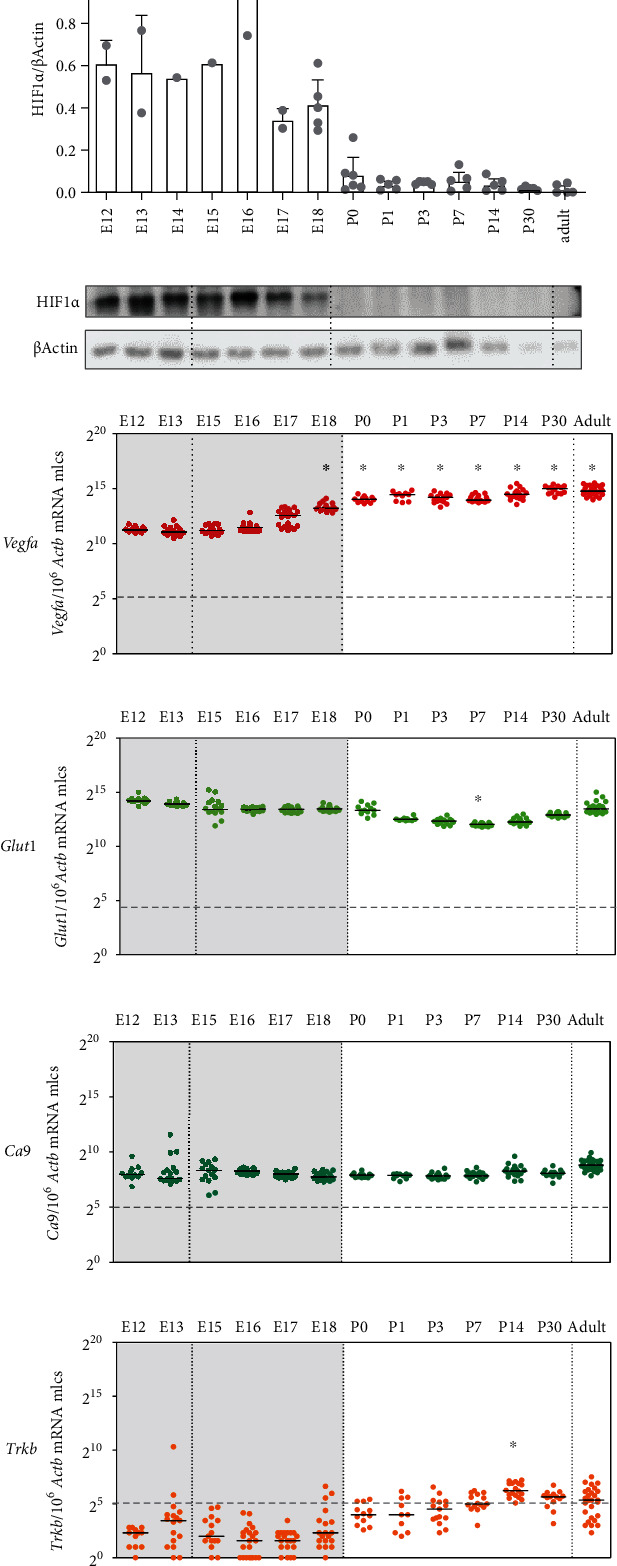
Expression patterns of HIF1*α* protein and of *bona fide* HIF-regulated genes (HRGs) in the developing mouse lung. Densitometric (a) and representative (b) HIF1*α* protein stabilization was detected by immunoblotting to show the hypoxic state of the lung during intrauterine development and its destabilization at birth. *β-Actin* served as a loading control, *n* = 1 − 6 mice per time point. (c–f) Absolute expression levels of *bona fide* HRGs were determined by qPCR (*n* = 10 − 23 mice per time point). One time point is represented by at least three different litters. Developmental stage is indicated by postconception days (E) or postnatal days (P) (for easier reference: 2^5^ = 32 molecules (mlcs), 2^10^ = 1 024 mlcs, and 2^15^ = 32 768 mlcs). Black bars represent the medians; the dashed line at 32 mlcs represents a physiological relevance threshold. The intrauterine period is indicated by the gray background color. Change points detected by the algorithms Sequen (#) or McDermott (∗) are indicated.

During lung development, the oxygen supply dramatically changes at birth with the onset of ventilation. This is reflected by a sudden destabilization of HIF1*α*protein from P0 onwards (Figures 1(a) and 1(b)). Against our expectations, this was not reflected by the expression levels of *bona fide* HRGs, though: *Glut1*, *Ca9*, and *Trkb* did not display developmental changes at all and *Vegfa* expression even increased from E18 onwards (Figures 1(c)–1(f)). Therefore, we asked the question whether expression changes in the HIF-PDH system compensated for the dramatic increase of oxygen after birth to stabilize HRG expression.

### 3.2. The Oxygen Sensing System Shows pO_2_-Related Expression Changes during Lung Development

**Figure 2:**
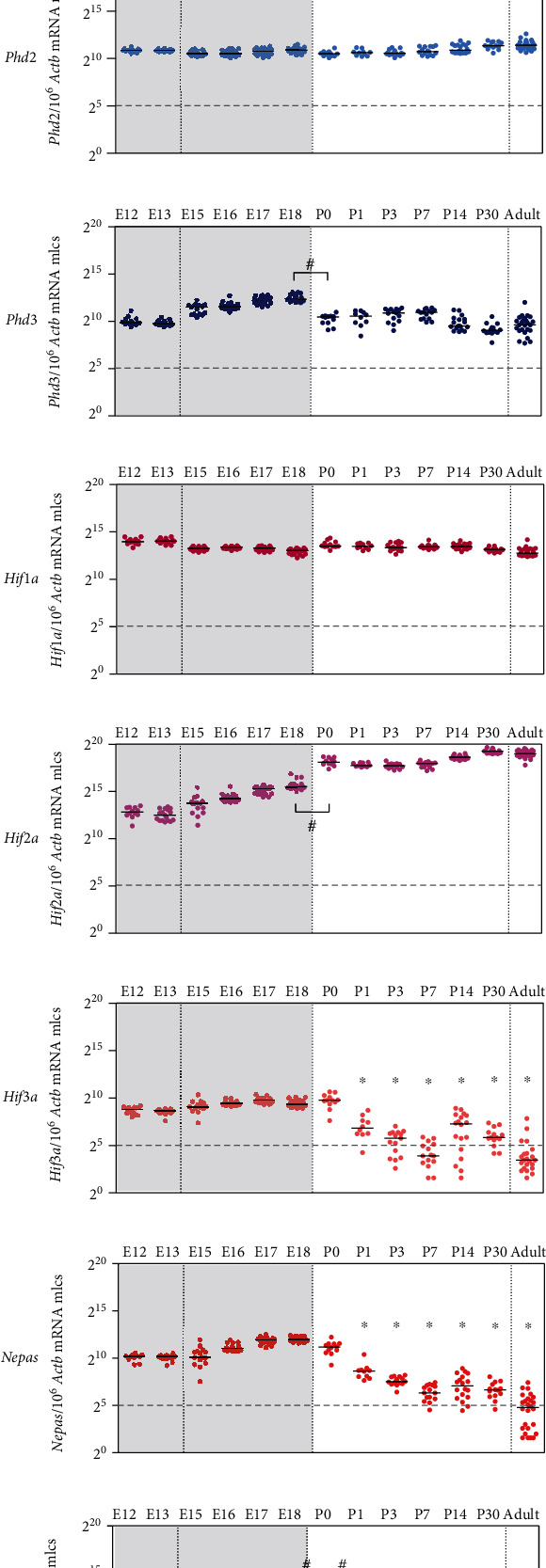
Expression patterns of the oxygen sensors (*Phds*) and effectors (*Hifs*) in the developing lung. (a–h) Absolute gene expression levels were determined by qPCR (*n* = 10 − 23 mice per time point). One time point is represented by at least three different litters. Developmental stage is indicated by postconception days (E) or postnatal days (P) (for easier reference: 2^5^ = 32 molecules (mlcs), 2^10^ = 1 024 mlcs, and 2^15^ = 32 768 mlcs). Black bars represent the medians; the dashed line at 32 mlcs represents a physiological relevance threshold. The intrauterine period is indicated by the gray background color. Change points detected by the algorithms Sequen (#) or McDermott (∗) are indicated.

To investigate the changes in the composition of the oxygen sensing system in the lung, gene expression of the oxygen sensors, prolyl hydroxylases *Phd1*, *Phd2*, and *Phd3*, and of the *Hifα* isoforms (*Hif1a* and *Hif2a*) and the three *Hif3a* isoforms (*Hif3a*, *Nepas*, and *Ipas*) was determined. Among the *Phds*, only *Phd3* showed a reduced expression level after birth (Figure 2(c)), while *Phd1* and *Phd2* remained constantly expressed (Figures 2(a) and 2(b)). *Hif1a* showed a constantly high expression throughout development (Figure 2(d)). As expected, *Hif1a* mRNA abundance differed from HIF1*α* protein levels due to the oxygen-mediated degradation of HIF1*α* protein (Figures 1(a) and 1(b)). In contrast, *Hif2a* showed a significant postnatal increase (Figure 2(e)). Analysis of *Hif3a* and its isoforms showed that those deserve special attention during the transition from low to high pO_2_: in adult organisms, the *Hif3a* isoforms were below the physiological relevance threshold (Figures 2(f)–2(h)). Isoform-specific quantification during lung development revealed a relatively constant intrauterine expression for *Hif3a* and *Nepas*, which significantly dropped stepwise during the postnatal periods of saccular and subsequent alveolar differentiation (Figures 2(f) and 2(g)). *Ipas* expression exhibited a slight increase, but rather low expression levels during gestation, followed by a drastic peak immediately after birth, before it also stepwise dropped in parallel to *Nepas* (Figure 2(h)). Our data strongly suggest that the HIF3*α* isoforms in combination with PHD3 play an important role in the transition from intrauterine to air-breathing life.

### 3.3. Colocalization Studies Point to the *Hif1a-Phd1* Axis as the Main Regulator of the HIF-PHD System in Lung Development, Complemented by the *Hif3a-Phd3* Axis *In Utero*

**Figure 3:**
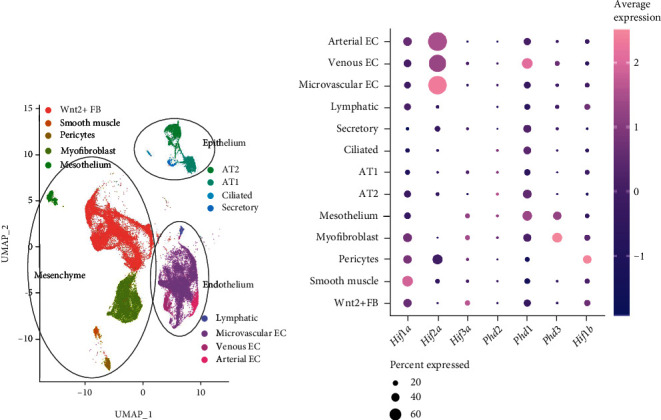
scRNA-seq analysis for cell type and average expression of HIF-PHD system genes in the mouse lung. (a) Cluster allocation for epithelial (9.5%), endothelial (30.7%), and mesenchymal (59.8%) lung cell types in the UMAP (Uniform Manifold Approximation and Projection) plot from integrated scRNA-seq datasets from all selected time points of the developing lung. Each color represents a cellular subtype as specified in the respective legend. (b) Average relative expression of the HIF-PHD system genes in each cell type, combined from all time points. EC: endothelial cell; AT1/2: alveolar type 1/2 cell; FB: fibroblast.

**Figure 4:**
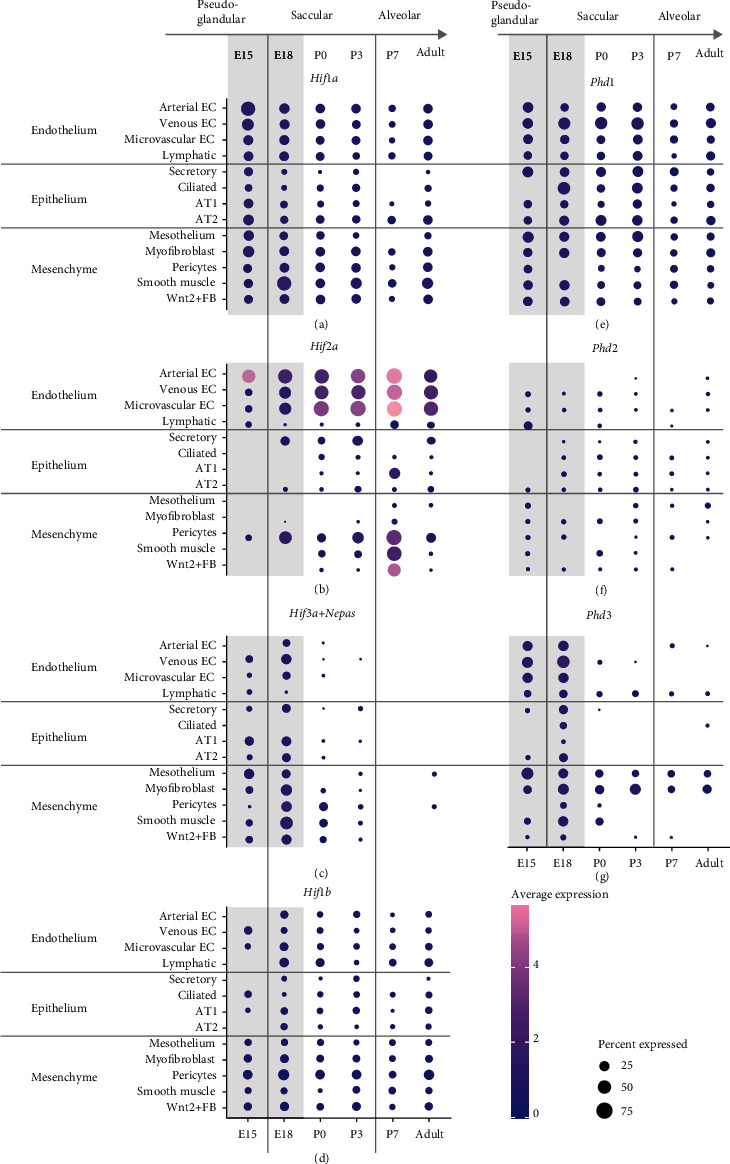
Expression pattern of the oxygen sensing system genes stratified by cell type and developmental age of the mouse lung by scRNA-seq data analysis. (a–g) Relative gene expression of the HIF-PHD system at the indicated time points stratified by selected cell types in lung epithelium, endothelium, or mesenchyme. Developmental stage is indicated by postconception days (E) or postnatal days (P); adult: P64. The intrauterine period is indicated by the gray background color. EC: endothelial cell; AT1/2: alveolar type 1/2 cell; FB: fibroblast.

**Figure 5:**
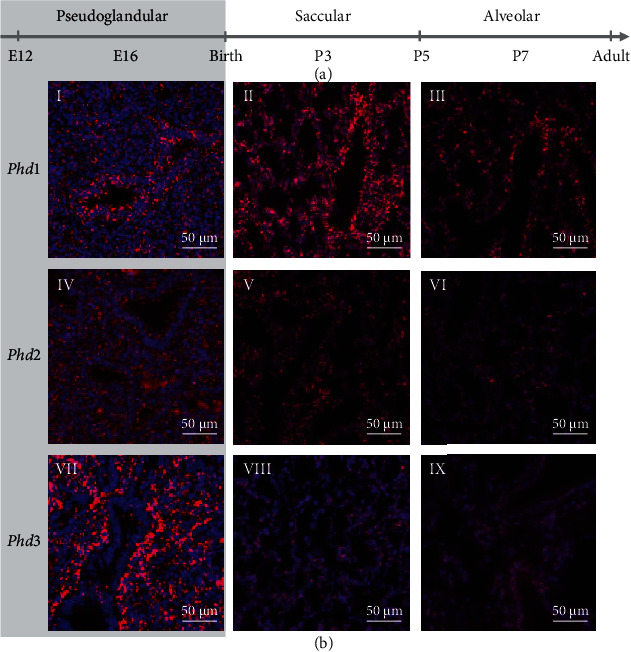
RNAscope *in situ* hybridization of the *Phds* during different stages of lung development. (a) Developmental stage is indicated by postconception days (E) or postnatal days (P). (b) Singleplex fluorescent staining of mouse lung sections at E16 (pseudoglandular phase: I, IV, and VII), P3 (saccular phase: II, V, and VIII), and P7 (alveolar phase: III, VI, and IX) in red (Opal 650) and DAPI in blue. Scale bars indicate 50 *μ*m. The intrauterine period is indicated by the gray background color.

**Figure 6:**
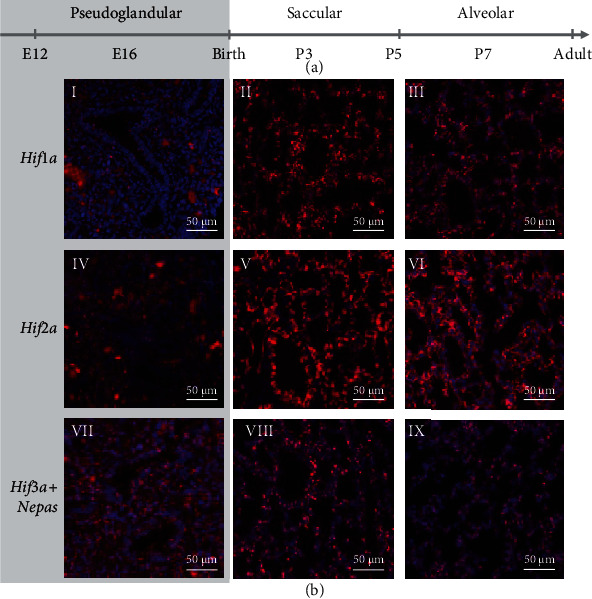
RNAscope *in situ* hybridization of the *Hifs* during different stages of lung development. (a) Developmental stage is indicated by postconception days (E) or postnatal days (P). (b) Singleplex fluorescent staining of mouse lung sections at E16 (pseudoglandular phase: I, IV, and VII), P3 (saccular phase: II, V, and VIII), and P7 (alveolar phase: III, VI, and IX) in red (Opal 650) and DAPI in blue. The probe for *Hif3a* detects both *Hif3a* and *Nepas*, but not *Ipas*. Scale bars indicate 50 *μ*m. The intrauterine period is indicated by the gray background color.

For physiological function, cellular coexpression is key. Therefore, we have taken advantage of published single-cell (sc)RNA-seq data from the normal mouse lungs at different gestational ages (E15, E18, P0, P3, P7, P14, and adult) [23] and analyzed them to stratify the genes of the oxygen sensing system by developmental age and lung parenchymal cell type (Figures 3 and 4). To complement the scRNA-seq data, lung tissues representative for the pseudoglandular, saccular, and alveolar phases were stained with the *in situ* hybridization technique RNAscope® (Figures 5 and 6).

The expression profile of the *Hifs* and *Phds* in pulmonary epithelium, endothelium, and mesenchyme showed distinct expression of the different components: *Phd1*, *Hif1a*, and *Hif1b* seemed to be the major components of the HIF-PHD system as they showed ubiquitous expression throughout lung development (Figures 4(a), 4(d), and 4(e), Figure 5(b) I-III, and Figure 6(b) I-III). In the case of *Hif1b*, this also means that HIF signaling is possible in each cell type and at all time points as HIF1*β* is indispensable for HRG induction [6]. *Hif2a* was preferentially expressed in endothelium and pericytes (Figures 4(b) and 6(b) IV-VI), showing predominant expression of all *Hif* and *Phd* transcripts. *Phd2* showed an overall low expression and was only slightly higher in lymphatic cells during the pseudoglandular phase (Figures 4(f) and 5(b) IV-VI). Both HRGs *Phd3* and *Hif3a+Nepas* showed high expression *in utero* (Figures 4(c) and 4(g), Figure 5(b) VII-IX, and Figure 6(b) VII-IX), and the loss of expression after birth (Figures 2(c), 2(f), and 2(g)) was confirmed here. In summary, these data showed that the HIF-PHD system had a specific composition in the different cellular subtypes during each period of lung development. Our colocalization studies point to the *Hif1a-Phd1* axis as the main regulator of the HIF-PHD system with an intrauterine complementation by the *Hif3a*-*Phd3* axis, while *Hif2a* seemingly has additional functions in endothelial cells and pericytes of the lung.

### 3.4. HRGs Are Strongly Inducible by HIF1*α* Stabilization in Lung *Ex Vivo* Organ Cultures of All Developmental Ages

**Figure 7:**
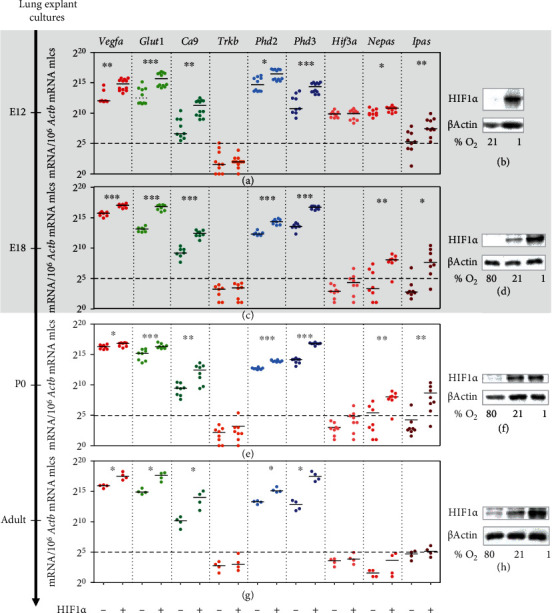
HRG expression with and without HIF1*α* protein stabilization in embryonic, fetal, neonatal, and adult lung *ex vivo* organ cultures. The lungs were dissected at E12 (a, b), E18 (c, d), P0 (e, f), and adult stage (g, h) and were cultured on transwell filters for 21 h at 80/21/1% O_2_ resulting in no (-) HIF1*α* or (+) stabilization (b, d, f, h). Absolute expression levels of *bona fide* HRGs were determined by qPCR (a, c, e, g). Developmental stage is indicated by postconception days (E) or postnatal days (P). The black bars represent the medians (for easier reference: 2^5^ = 32 molecules (mlcs), 2^10^ = 1 024 mlcs, and 2^15^ = 32 768 mlcs). The intrauterine period is indicated by the gray background color. Statistical significance (Mann-Whitney *U* test, *n* = 4 − 12 per condition) is indicated by ^∗^*p* < 0.05 and ^∗∗∗^*p* < 0.001. (b, d, f, h) HIF (de)stabilization was confirmed by immunoblotting of 4 pooled lung *ex vivo* organ culture lysates for each set of experiments. *β-Actin* served as a loading control.

Taken the small changes of the pulmonary HRGs from intrauterine to air-breathing life, we asked whether their capacity to respond to different oxygen levels changed during development. Since we did not succeed in transplacentally stabilizing HIF in the embryo for longer than 8 hours, we established lung *ex vivo* organ cultures instead to determine the response of HRGs under defined high vs. low oxygen levels, resulting in two conditions: presence and absence of HIF1*α* protein. Under these two extreme *ex vivo* conditions, lung organ cultures of all gestational ages did strongly react to Hif1*α* stabilization (Figures 7(a), 7(c), 7(e), and 7(g)), showing that the hypoxic inducibility already existed from E12 onwards. Embryonic lung *ex vivo* organ cultures required only incubation at 21% O_2_ to destabilize HIF1*α* protein, while fetal, neonatal, and adult lung cultures required 80% O_2_ to achieve this effect (Figures 7(b), 7(d), 7(f), and 7(h)). Concerning the extent of induction, it was not so much the hypoxic but more the basal expression level in the absence of HIF1*α* that changed throughout development. In this setting, the *Hif3a* isoform did not seem to be a *bona fide* HRG since it did not show any induction in the *ex vivo* organ cultures. In summary, this *ex vivo* approach showed that a very strong hypoxic stimulus activated HRG transcription in the lung at all developmental stages.

### 3.5. The *In Vivo* Hypoxic Response Is Restricted to Selected Downstream HRGs in the Adult Lung

**Figure 8:**
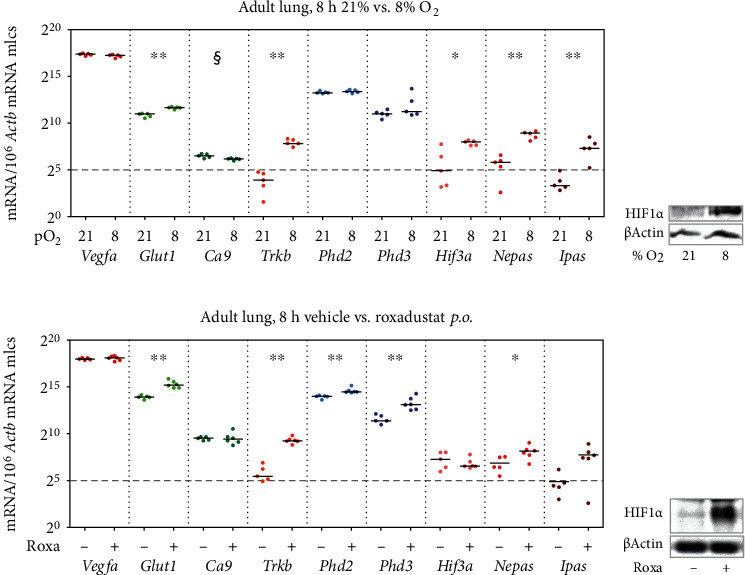
Effects of systemic hypoxia or roxadustat treatment on HRG expression *in vivo*. (a, c) Absolute expression quantification of *bona fide* HRGs in adult mice exposed to 21% vs. 8% O_2_ for 8 h or treated with vehicle or roxadustat for 8 h, respectively. Statistically significant induction (Mann-Whitney *U* test; systemic hypoxia: *n* = 5 per condition, roxadustat: *n* = 5 − 6 per condition) in comparison to the respective controls is indicated by ^∗^*p* < 0.05 and ^∗∗^*p* < 0.01, repression by §. The black bars represent the medians. (b, d) HIF (de)stabilization was confirmed by immunoblotting. *β-Actin* served as a loading control.

With the use of the *ex vivo* lung organ cultures, we established the maximal response of the HRGs to extreme changes in oxygen. This determined the range in which the oxygen sensing system was able to react. To classify the response of HRGs to physiological oxygen changes within this range, we analyzed the expression of HRGs in mice exposed to acute systemic hypoxia (8 h, 8% O_2_). Here, we observed repressed *Ca9* expression and no change in expression of *Vegfa*, *Phd2*, or *Phd3*. However, expression of *Glut1*, *Trkb*, and all *Hif3a* isoforms was significantly induced in the mouse lung (Figure 8(a)). The extent of induction was small in the case of the well-expressed *Glut1* and higher in low-expressed transcripts (*Trkb*, *Hif3a*, *Nepas*, and *Ipas*). Similar results were observed for HRGs in rats exposed to systemic 21% vs. 8% O_2_ (Supplementary Figure S1). In an additional approach, we treated mice with roxadustat (or vehicle) for 8 h to stabilize HIF and analyzed the resulting HRG expression in the lungs. The reactions were slightly different from systemic hypoxia. The *x*-fold induction was higher, and additional genes were significantly induced (*Phd2* and *Phd3*), while among the *Hif3a* isoforms, only *Nepas* was significantly induced (Figure 8(c)). In summary, our data suggests that the lung has the full capacity to react to a drop in oxygen levels but that only parts of this maximal *ex vivo* capacity are used *in vivo*.

## 4. Discussion

Our study extends current knowledge on the adaption of the oxygen sensing system during murine lung development. To the best of our knowledge, our data describe for the first time changes in the HIF-PHD system from intrauterine to postnatal development of the lung. Our colocalization studies point to the *Hif1a-Phd1* axis as the main regulator of the HIF-PHD system, complemented by the *Hif3a-Phd3* axis during gestation (Figures 4–6). In line with suppressed Hif1*α* stabilization after birth (Figures 1(a) and 1(b)), the HRGs *Phd3* and *Hif3a+Nepas* show a (stepwise) drop in expression from birth onwards (Figures 4(c) and 4(g), Figure 5(b) VII-IX, and Figure 6(b) VII-IX). We observe low levels of *Phd2* expression (Figures 4(f) and 5 IV-VI) in all periods of lung development, which is surprising as PHD2 was described as the major oxygen sensor in the adult [27] and as a pivotal driver in embryonic development as demonstrated by *Phd2* knockout experiments [10]. PHD2 is dominant in inhibiting HIF*α* in normoxia in all human cells analyzed so far [28]. PHD3 seems to be responsible for fine-tuning of the HIF response via a negative feedback loop when PHD2 is already compromised in prolonged hypoxia [29]. The optimal oxygen level for PHD3 activity seems to be lower than for PHD2 [30]. That would mean that PHD3 remains active during chronic intermediate hypoxia when PHD2 is already inactivated. This may explain why we have found *Phd3* strongly expressed in the intrauterine period of lung development (Figure 2(c)) to guarantee enough PHD activity in a low pO_2_ environment. After birth with a higher pO_2_ level and normal PHD1/2 activity, PHD3 may no longer be required at such high amounts. In baboons, no change in PHD1 protein occurs during the transition from intrauterine to air-breathing life, but an increase in PHD2 and PHD3 [31]. This is different from our finding of *Phd1* and *Phd2* being unchanged and *Phd3* diminishing after birth (Figures 2(a)–2(c)). This might be due to species differences, however, with the pO_2_ increase by lung ventilation happening already during the saccular phase in mice, but only during the alveolar phase in baboons. In human cell lines, PHD3 was found to preferentially act on HIF2*α* (rather than HIF1*α*, HIF3*α* was not tested) and to preferentially hydroxylate the C-terminal proline in the ODDD [32] which also exists in HIF3*α*. Therefore, a specific action of PHD3 on HIF3*α* seems feasible. *Hif3a* and *Nepas* expression remained stable during intrauterine development, but significantly decreased after birth with a pattern that suggested a stepwise adaptation of its expression levels according to the maturation of the bronchoalveolar tissue structures (Figures 2(f) and 2(g)). In the lung, neonatal *Hif3a+Nepas* was localized in endothelial cells by immunohistochemistry [13]. We have observed not only endothelial expression but also mesenchymal and some epithelial expression which completely disappeared by beginning of the alveolar phase (Figure 4(c)). This might indicate that the function of HIF3 is terminated with the presence of functional alveoli. The importance of the intrauterine PHD3-HIF3 axis is underlined by the lung phenotype of the *Hif3a* knockout mice. These mice are viable but display pulmonary endothelial hyperplasia with increased endothelin 1 expression as well as disturbed and preterm alveolar septation [13]. Endothelial cells from the *Hif3a+Nepas* knockout lungs express more proangiogenic factors but show reduced angiogenesis. This effect can be attributed to lacking repression of the transcription factors HIF2*α* and ETS protooncogene 1 by HIF3*α*. Subsequently, increased VE-cadherin partly inhibits the VEGF signaling pathway and thereby normal angiogenesis [33].

Apart from *Phd3*, only *Hif3a/Nepas/Ipas* showed pO_2_-related expression changes during lung development (Figures 2(f)–2(h)). Additionally, we observed a hypoxic induction of *Nepas* and *Ipas* in our *ex vivo* and *in vivo* models (Figures 7(a), 7(c), 7(e), and 7(g) and Figures 8(a) and 8(c)), which is in line with the findings from Yamashita et al. [13] showing that *Ipas* and *Nepas* expression was induced in mice by systemic hypoxia (<10% O_2_). For *Hif3a*, we found an increase in expression only in the *in vivo* model of systemic hypoxia (Figure 8(a)) but none in the other *ex vivo* or *in vivo* models (Figures 7(a), 7(c), 7(e), 7(g) and 8(c)). Our data suggest that only *Nepas* and *Ipas* are directly regulated by hypoxia. Interestingly, due to this feature, HIF3*α* protein is considered a sensitive and rapidly reacting component of the HIF signaling pathway in protection against hypoxic damage [34].

Notably, we observed a peak in *Ipas* expression right after birth at P0 (Figure 2(h)). This peak, however, is not necessarily a result of hypoxic induction as observed in our *ex vivo* and *in vivo* models (Figures 7 and 8(a)). It might only be functionally associated but not causally related with the pO_2_ increase at birth and deserves further investigation. Given that IPAS acts as a dominant negative regulator of the HIF signaling pathway [15, 16], the sharp and short-termed increase in *Ipas* expression at P0 might be necessary to shut down HIF-mediated transcriptional responses immediately after birth by scavenging any remaining HIF*α*, perhaps even with some preference for the HIF1*α* isoform as the interaction with HIF2*α* is far weaker and has only been shown for the human IPAS orthologue HIF3*α*-4 [15].

We observed that HIF1*α* protein is completely destabilized with the onset of lung ventilation but this does not lead to an expression change of *bona fide* HRGs (Figure 1). The observed HIF1*α* protein destabilization after birth is in line with findings in baboons [31]. In lung *ex vivo* organ cultures, we were able to show that, with a strong hypoxic stimulus, the HIF system was functional at every developmental stage (Figure 7). In our *in vivo* approaches with systemic hypoxia or roxadustat treatment, only parts of the HRGs strongly inducible in lung *ex vivo* organ cultures reacted to HIF1*α* stabilization (Figure 8 and Figure S1). This might be due to a weaker hypoxic stimulus *in vivo*, showing a limited HRG response. Therefore, the first hypothesis is that HIF1*α* destabilization at the onset of lung ventilation is not strong enough to result in major HRG expression changes. A second hypothesis is that HIF1*α* protein is efficiently degraded (Figure 1), while HIF2*α* and HIF3a/IPAS/NEPAS are not. Asikainen et al. showed in baboons that HIF1*α* protein disappeared after birth while HIF2*α* protein diminished before birth and was then again stabilized at day 2 after birth [31]. *Hif2a* knockout studies implied that HIF2*α* plays an important role in the development of alveolar structures, the differentiation of alveolar type 1 and type 2 (AT1/2) cells, and surfactant production [11, 35]. *Hif2a* showed an incremental increase from the fetal period onwards which stabilized at very high postnatal levels and which persisted into adulthood (Figure 2(e)). After birth, the high *Hif2a* mRNA level might be a compensating mechanism for the increased degradation efficiency of the PHDs to guarantee that some of the HIF2*α* protein will stay functional. In our scRNA-seq data, *Hif2a* expression is highest in endothelial cells and pericytes (Figure 3(b)). Therefore, the stabilizing effect is probably restricted to these cell types. As endothelial cells account for 30% of the lung parenchymal cells, HIF2*α* stabilization in these cells might compensate for the degradation of HIF1*α* protein after birth and therefore stabilize HRG expression under these changing oxygen conditions. The localization we observed is slightly different from the immunohistochemistry data from Wiesener et al. showing HIF2*α* protein expression in the hypoxic rat lungs in the vascular endothelium and in AT2 cells, but not in pericytes [36]. In our scRNA-seq data, AT2 cells express very little *Hif2a* (Figure 3(b)). Besides the increase in *Hif2a* mRNA, we observe changes in the composition of the PHD-HIF system, which could influence the continuous expression of the HRGs before and after birth. *Phd3* levels dropped immediately after birth (Figure 2(c)). With PHD3 preferentially hydroxylating HIF2*α* [32], the reduced expression of *Pdh3* might also support postnatal HIF2*α* stabilization. Due to the lack of a specific antibody against mouse HIF2*α* protein, this hypothesis could not be tested so far. If postnatal HIF2*α* activity despite high environmental oxygen levels is actually key for the correct alveolarization of the lung, this might explain why PHD inhibition ameliorates the lung hypoplasia observed in mouse models of hyperoxia-induced lung injury [37, 38] or in primate models of bronchopulmonary dysplasia [39, 40]. Our data support these previous concepts of a selective use of HIF-stabilizing agents in order to promote proper vascularization of the interstitial pulmonary tissue in very premature infants.

## 5. Conclusions

This study contributes to a detailed map of the oxygen sensing system with a cellular resolution and in a developmental context. Understanding the interplay of the different components of the HIF-PHD system during the critical transition from saccular to alveolar phases of lung development (ideally at a cellular resolution level) might help to counteract prematurity-associated diseases like bronchopulmonary dysplasia. In this context, the developmental changes in the HIF3*α*-PHD3 axis deserve particular attention.

## Data Availability

The data used to support the findings of this study are available from the corresponding author upon request. scRNA-seq data was obtained and is available from the NCBI GEO database (Accession GSE165063 and GSE160876).

## Supplementary Materials

Supplementary 1 Supplementary Table S1: primers and probes used for detection of the respective mRNA in mouse or rat transcripts.

Supplementary 2 Supplementary Table S2: point estimates and confidence intervals for longitudinal gene expression in normal lung development.

Supplementary 3 Supplementary Figure S1: Supplementary Figure S2: effects of systemic hypoxia on HRG expression in rats.
