# Supplementary material for: Adaptation of the Oxygen Sensing System during Lung Development
Source: Oxid Med Cell Longev. 2022 Feb 18;2022:9714669. doi: 10.1155/2022/9714669 (PMC8886745; doi:10.1155/2022/9714669)
Supplement: Supplementary 1 — Supplementary Table S1: primers and probes used for detection of the respective mRNA in mouse or rat transcripts. [file 9714669.f1.pdf]

Supplementary Table S1: Primers and probes used for detection of the respective mRNA in mouse (m) or rat (r) transcripts.

	**Forward primer (5'→3')**	**Probe (5'→3')**	**Reverse primer (5'→3')**	**NCBI accession no.**
mPhd1 (mEgln2)	GGGGGAAAGTGGTATGGGCTG	TGGCGAGGCCATGGTGGC GTG	CCGAGCCCATTGCCTGGATAA	NM_001357767.1
mPhd2 (mEgln1)	CTGACCGGCGTAACCCTCAT	GCCAGCATACGCCACAAGG TACGCA	TCTCGCTCGCTCATCTGCAT	NM_053207.2
mPhd3 mEgln3)	GAGCCGGCTGGGCAAATACT	GGAGCGGTCCAAGGCAAT GGTGGCT	TTGGGGTTGTCCACATGGCG	NM_028133.2
mHif1a	GGGGAGGACGATGAACATCAA GTC	GGAAGGTGCTTCACTGCAC GGGCCA	CCCACACTGAGGTTGGTTACTGTT	NM_010431.2
mHif2a (mEpas1)	CCATGCCCTGGATTCGGAGAA	ACTTGTGCACCAAGGGGCA GGTGGT	CGTGTTTGGCTAGCATCCGGTA	NM_010137.3
mHif3a	GGCACACAACCTAGGGGAGAA	TCAGCGCGCACCTGGACAA GGCCTC	TTCCACCTGGTTCCACTCCC	NM_016868.3
mNepas	GGCATCGAGGGCTCTGGAAC	TCAGCGCGCACCTGGACAA GGCCTC	TCAGGTAGCAGGCGTCCAGT	NM_001162950.1
mIpas	GTACCAGCTGGCGCACACTC	TCAGCGCGCACCTGGACAA GGCCTC	GGAGGAACAGAGGTCCACTGACT	AF416641
mVegfa	TGTGCAGGCTGCTGTAACGAT	AAGCCCTGGAGTGCGTGCC CACGTC	GTGCTGGCTTTGGTGAGGTTTG	NM_009505.4
mGlut1 (mSlc2a1)	TGTGCTCATGACCATCGCCCT	GGCCTTGCTGGAACGGCTG CCTTGG	AAGCCAAAGATGGCCACGATGC	NM_011400.3
mTrkb (mNtrk2)	CGAGTGCTACAACCTCTGCCC	GGCTGTGAAGACGCTGAA GGACGCC	GTCCTTGCGTGCATTGTCGC	NM_001025074.2
mCa9 (mCar9)	GGAGGCCTGGCAGTTTTGG	TGCCTTTCTGCAGGAGAGC CCAGAAGA	GACAGCAACTGTTCGTAAGCACT	NM_139305.2
rPhd2 (rEgln1)	ATGAACAAGCACGGCATCTG		TTGGGTTATCGACGTGACGG	XM_039098215.1
rPhd3 (rEgln3)	ATGGTGATGGCCGCTGTATC		GACTTCACACCACCGTCAGT	NM_019371.2
rVegfa	GTACCTCCACCATGCCAAGT		CTGCATGGTGATGTTGCTT	NM_001110334
rTrkb (rNtrk2)	GTGGAGGAAGGGAAGTCTGTG		CAGTGGTGGTCTGAGGTTGGA	M55293.1
rCa9 (rCar9)	ATCACCCAGGCTCAGAACAC		TCGGTAGTAGCGACTGAGGT	NM_001107956.1
rActin	ATGGTGGGTATCAGAA		GGGGTGTTGACTCAAA	NM_031144
