# Supplementary material for: Adaptation of the Oxygen Sensing System during Lung Development
Source: Oxid Med Cell Longev. 2022 Feb 18;2022:9714669. doi: 10.1155/2022/9714669 (PMC8886745; doi:10.1155/2022/9714669)
Supplement: Supplementary 2 — Supplementary Table S2: point estimates and confidence intervals for longitudinal gene expression in normal lung development. [file 9714669.f2.pdf]

Supplementary Table S2: Point estimates and confidence intervals for longitudinal gene expression in normal lung development. Seq: Sequen, md: McDermott

Phd1							
**contrast.seq**	**estimate.seq**	**conf.low.seq**	**conf.high.seq**	**contrast.md**	**estimate.md**	**conf.low.md**	**conf.high.md**
2 - 1	0,414271501	-0,016855741	0,845398743	C 1	0,414271501	-0,004540537	0,833083539
3 - 2	-0,263374316	-1,167682609	0,640933977	C 2	-0,091951626	-0,953164571	0,769261319
4 - 3	-0,153497537	-0,536473942	0,229478868	C 3	-0,214102018	-0,807465507	0,379261471
5 - 4	-0,201825735	-0,550725197	0,147073728	C 4	-0,344560413	-0,787953711	0,098832885
6 - 5	-0,001787798	-0,3640596	0,360484004	C 5	-0,263178456	-0,647496534	0,121139621
7 - 6	0,028456238	-0,8057958	0,862708276	C 6	-0,187548721	-0,833327411	0,458229969
8 - 7	-0,323833157	-0,813675746	0,166009432	C 7	-0,492309127	-1,107307205	0,122688951
9 - 8	-0,193235097	-0,660304712	0,273834517	C 8	-0,647082574	-1,194254469	-0,099910678
10 - 9	0,037305856	-0,380227253	0,454838964	C 9	-0,541900924	-1,048988424	-0,034813423
11 - 10	-0,106009442	-0,505766959	0,293748075	C 10	-0,596464076	-1,057485372	-0,13544278
12 - 11	-0,112700714	-0,520319438	0,294918011	C 11	-0,648162782	-1,100964013	-0,19536155
13 - 12	-0,053935364	-0,444956702	0,337085974	C 12	-0,654338783	-1,022958865	-0,285718701
Phd2							
**contrast.seq**	**estimate.seq**	**conf.low.seq**	**conf.high.seq**	**contrast.md**	**estimate.md**	**conf.low.md**	**conf.high.md**
2 - 1	-0,007895973	-0,273280706	0,257488759	C 1	-0,007895973	-0,256638701	0,240846754
3 - 2	-0,26352081	-1,03191853	0,504876909	C 2	-0,26678811	-0,979607551	0,446031331
4 - 3	-0,008397143	-0,244143857	0,227349571	C 3	-0,184234761	-0,665137667	0,296668144
5 - 4	0,12600959	-0,088759962	0,340779143	C 4	0,003186416	-0,33877484	0,345147672
6 - 5	0,193976461	-0,029027712	0,416980633	C 5	0,196393742	-0,085542836	0,47833032
7 - 6	-0,373946509	-1,110526311	0,362633294	C 6	-0,212755419	-0,738965211	0,313454374
8 - 7	0,132134673	-0,169397022	0,433666369	C 7	-0,058984601	-0,547008583	0,429039381
9 - 8	-0,038102629	-0,325616391	0,249411134	C 8	-0,092479058	-0,53363628	0,348678165
10 - 9	0,194700624	-0,062316102	0,45171735	C 9	0,111922167	-0,290593816	0,514438149
11 - 10	0,245032383	-0,001042398	0,491107164	C 10	0,346329027	-0,018896439	0,711554494
12 - 11	0,310983028	0,060066665	0,561899392	C 11	0,621892042	0,276353498	0,967430585
13 - 12	0,060857928	-0,178750969	0,300466826	C 12	0,636926346	0,342877828	0,930974863
Phd3							
**contrast.seq**	**estimate.seq**	**conf.low.seq**	**conf.high.seq**	**contrast.md**	**estimate.md**	**conf.low.md**	**conf.high.md**
2 - 1	-0,181568371	-0,687994329	0,324857586	C 1	-0,181568371	-0,657179007	0,294042264
3 - 2	1,086209699	-0,33225838	2,504677779	C 2	1,008394683	-0,308074964	2,32486433
4 - 3	0,312183068	-0,13197858	0,756344716	C 3	0,968812164	0,088999825	1,848624502
5 - 4	0,596689336	0,192049934	1,001328737	C 4	1,237595844	0,613162822	1,862028866
6 - 5	0,238116771	-0,182036935	0,658270477	C 5	1,173508979	0,657176413	1,689841544
7 - 6	-2,317388775	-3,67164394	-0,963133611	C 6	-1,35622904	-2,334541005	-0,377917075
8 - 7	0,078786549	-0,489320648	0,646893746	C 7	-1,138342077	-2,045778959	-0,230905195
9 - 8	0,389707882	-0,151988753	0,931404517	C 8	-0,65900096	-1,478388888	0,160386968
10 - 9	0,120057773	-0,364178062	0,604293608	C 9	-0,469330409	-1,216920414	0,278259595
11 - 10	-0,998033935	-1,461654449	-0,53441342	C 10	-1,422523859	-2,10055252	-0,744495198

12 - 11	-0,625473516	-1,098217311	-0,152729722	C 11	-1,901680636	-2,543988873	-1,259372398
13 - 12	0,477313092	0,017400054	0,93722613	C 12	-1,283502312	-1,832258242	-0,734746382
Hif1a							
**contrast.seq**	**estimate.seq**	**conf.low.seq**	**conf.high.seq**	**contrast.md**	**estimate.md**	**conf.low.md**	**conf.high.md**
2 - 1	0,052010952	-0,225687812	0,329709717	C 1	0,052010952	-0,214151134	0,318173038
3 - 2	-0,997761459	-1,622382235	-0,373140683	C 2	-0,973756404	-1,559689036	-0,387823772
4 - 3	0,144136419	-0,092288897	0,380561735	C 3	-0,473367642	-0,861535929	-0,085199355
5 - 4	-0,124413711	-0,339801781	0,090974359	C 4	-0,432478367	-0,714187207	-0,150769526
6 - 5	-0,320686163	-0,544331027	-0,097041299	C 5	-0,645044938	-0,885536338	-0,404553538
7 - 6	0,67711792	0,089918541	1,264317299	C 6	0,151061854	-0,288601624	0,590725333
8 - 7	-0,205601684	-0,508936242	0,097732874	C 7	-0,070302806	-0,488969721	0,348364108
9 - 8	-0,056878898	-0,345219626	0,231461829	C 8	-0,12155748	-0,496198107	0,253083147
10 - 9	0,059578311	-0,198179173	0,317335794	C 9	-0,048955154	-0,393091624	0,295181316
11 - 10	0,024979329	-0,221804672	0,271763331	C 10	-0,019238229	-0,331387964	0,292911506
12 - 11	-0,338831603	-0,590470155	-0,087193052	C 11	-0,356068167	-0,657440124	-0,05469621
13 - 12	-0,219179303	-0,457777596	0,019418991	C 12	-0,54858996	-0,798563294	-0,298616625
Hif2a							
**contrast.seq**	**estimate.seq**	**conf.low.seq**	**conf.high.seq**	**contrast.md**	**estimate.md**	**conf.low.md**	**conf.high.md**
2 - 1	-0,277584384	-0,756509039	0,201340272	C 1	-0,277584384	-0,757225219	0,202056451
3 - 2	1,138846331	0,514663188	1,763029474	C 2	1,023983828	0,431211083	1,616756572
4 - 3	0,787682571	0,362265753	1,213099389	C 3	1,462581003	1,024576095	1,90058591
5 - 4	0,836385058	0,448814933	1,223955183	C 4	1,81143906	1,440130807	2,182747312
6 - 5	0,478892819	0,076502051	0,881283587	C 5	1,853087968	1,50000479	2,206171145
7 - 6	2,496001607	1,912833986	3,079169227	C 6	4,016932297	3,529908282	4,503956312
8 - 7	-0,297379183	-0,841476124	0,246717759	C 7	3,311051525	2,815013164	3,807089886
9 - 8	-0,12200876	-0,64080541	0,39678789	C 8	2,930366865	2,50581997	3,354913759
10 - 9	0,124742525	-0,339080868	0,588565918	C 9	2,74772825	2,339017481	3,156439019
11 - 10	0,797333017	0,353255927	1,241410107	C 10	3,284200991	2,910922967	3,657479014
12 - 11	0,608847404	0,156069817	1,06162499	C 11	3,557164202	3,161559358	3,952769046
13 - 12	-0,275611011	-0,699465145	0,148243123	C 12	3,019446356	2,723431381	3,31546133
Hif3a							
**contrast.seq**	**estimate.seq**	**conf.low.seq**	**conf.high.seq**	**contrast.md**	**estimate.md**	**conf.low.md**	**conf.high.md**
2 - 1	-0,092844141	-1,178417759	0,992729477	C 1	-0,092844141	-1,176669053	0,990980771
3 - 2	0,485152923	-1,041463293	2,011769139	C 2	0,446734657	-1,009946867	1,903416181
4 - 3	0,384374625	-0,579926535	1,348675786	C 3	0,678813377	-0,380411998	1,738038752
5 - 4	0,304223548	-0,574285088	1,182732183	C 4	0,756765799	-0,11917262	1,632704217
6 - 5	-0,339368849	-1,251495467	0,572757768	C 5	0,234729343	-0,585631132	1,055089817
7 - 6	0,221095263	-1,191118764	1,63330929	C 6	0,413750478	-0,756482796	1,583983752
8 - 7	-2,836004881	-4,06933975	-1,602670012	C 7	-2,464330723	-3,640868895	-1,28779255
9 - 8	-1,517055074	-2,693043782	-0,341066366	C 8	-3,788859959	-4,803204497	-2,774515421
10 - 9	-1,187655835	-2,238999575	-0,136312094	C 9	-4,579082931	-5,548584605	-3,609581257

11 - 10	2,185218279	1,178633282	3,191803276	C 10	-1,959141589	-2,843856747	-1,074426432
12 - 11	-0,377494611	-1,403822943	0,648833722	C 11	-2,136269446	-3,061788236	-1,210750657
13 - 12	-2,058741123	-3,022419136	-1,09506311	C 12	-4,037601241	-4,735428953	-3,33977353
Nepas							
**contrast.seq**	**estimate.seq**	**conf.low.seq**	**conf.high.seq**	**contrast.md**	**estimate.md**	**conf.low.md**	**conf.high.md**
2 - 1	0,023202904	-0,894579611	0,940985419	C 1	0,023202904	-0,881637298	0,928043106
3 - 2	0,532384962	-1,058635319	2,123405242	C 2	0,541986163	-0,981256574	2,065228901
4 - 3	0,967908246	0,15263687	1,783179622	C 3	1,325126399	0,253935016	2,396317782
5 - 4	0,766258169	0,02352628	1,508990058	C 4	1,649675768	0,814675439	2,484676097
6 - 5	0,132490205	-0,638694469	0,903674879	C 5	1,383968374	0,633928257	2,134008491
7 - 6	-0,809531151	-2,260596432	0,641534131	C 6	0,326367421	-0,842439797	1,495174639
8 - 7	-2,515531667	-3,558283326	-1,472780008	C 7	-2,222354154	-3,361251618	-1,083456689
9 - 8	-0,963381847	-1,957653292	0,030889598	C 8	-3,012114582	-4,011820994	-2,012408171
10 - 9	-1,223507408	-2,112350768	-0,334664047	C 9	-3,919665915	-4,85843809	-2,98089374
11 - 10	0,617895627	-0,233107101	1,468898355	C 10	-2,929650106	-3,784525863	-2,074774349
12 - 11	-0,344916125	-1,212639099	0,52280685	C 11	-2,974942925	-3,838874439	-2,11101141
13 - 12	-2,27226578	-3,093615819	-1,450915741	C 12	-5,028002384	-5,705656499	-4,350348269
Ipas							
**contrast.seq**	**estimate.seq**	**conf.low.seq**	**conf.high.seq**	**contrast.md**	**estimate.md**	**conf.low.md**	**conf.high.md**
2 - 1	2,259439807	0,852166519	3,666713094	C 1	2,259439807	0,842892922	3,675986691
3 - 2	0,196194694	-1,128223898	1,520613287	C 2	1,16452604	-0,022368507	2,351420587
4 - 3	0,968842947	-0,265096746	2,202782641	C 3	1,727138973	0,754809026	2,699468921
5 - 4	0,379829734	-0,744421266	1,504080735	C 4	1,522398594	0,591321908	2,453475279
6 - 5	-0,095078208	-1,261870605	1,07171419	C 5	1,055571892	0,115261631	1,995882153
7 - 6	5,104896655	3,74607185	6,46372146	C 6	5,9694603	4,839117401	7,099803198
8 - 7	-4,164782729	-5,742651062	-2,586914396	C 7	1,192425232	-0,029688099	2,414538563
9 - 8	-1,613036898	-3,117475324	-0,108598472	C 8	-0,514503417	-1,527246379	0,498239546
10 - 9	-2,326538509	-3,672149144	-0,980927873	C 9	-2,786692973	-3,793769739	-1,779616207
11 - 10	1,526339348	0,220905282	2,831773414	C 10	-0,994109073	-1,941224453	-0,046993693
12 - 11	-1,154006026	-2,48397776	0,175965709	C 11	-2,0509895	-3,081477253	-1,020501747
13 - 12	-0,652027507	-1,873628414	0,5695734	C 12	-2,550283746	-3,32643049	-1,774137002
Glut1							
**contrast.seq**	**estimate.seq**	**conf.low.seq**	**conf.high.seq**	**contrast.md**	**estimate.md**	**conf.low.md**	**conf.high.md**
2 - 1	-0,325840708	-0,713313663	0,061632247	C 1	-0,325840708	-0,71506287	0,063381455
3 - 2	-0,398218112	-0,837991585	0,04155536	C 2	-0,53304875	-0,94440179	-0,121695711
4 - 3	-0,185054676	-0,529222884	0,159113532	C 3	-0,536382261	-0,851545845	-0,221218677
5 - 4	-0,025023478	-0,338578213	0,288531257	C 4	-0,382611652	-0,663285361	-0,101937944
6 - 5	0,073763036	-0,251756042	0,399282114	C 5	-0,21649408	-0,490686531	0,057698372
7 - 6	-0,100380915	-0,524884066	0,324122237	C 6	-0,278069452	-0,634250274	0,07811137

8 - 7	-0,883947652	-1,324109893	-0,44378541	C 7	-1,133738854	-1,505656644	-0,761821064
9 - 8	-0,212898154	-0,632589386	0,206793077	C 8	-1,258063661	-1,572032866	-0,944094455
10 - 9	-0,278317614	-0,653572925	0,096937698	C 9	-1,404416555	-1,710823984	-1,098009126
11 - 10	0,311156058	-0,048123549	0,670435666	C 10	-0,959929811	-1,240204802	-0,67965482
12 - 11	0,600578493	0,234283194	0,966873792	C 11	-0,261176678	-0,565155404	0,042802048
13 - 12	0,643428676	0,302456213	0,98440114	C 12	0,401496595	0,175916088	0,627077103
Ca9							
**contrast.seq**	**estimate.seq**	**conf.low.seq**	**conf.high.seq**	**contrast.md**	**estimate.md**	**conf.low.md**	**conf.high.md**
2 - 1	0,156902932	-0,421868042	0,735673906	C 1	0,156902932	-0,425107987	0,73891385
3 - 2	-0,140515189	-0,790652017	0,50962164	C 2	-0,075589838	-0,683391875	0,5322122
4 - 3	0,179883396	-0,334200677	0,693967468	C 3	0,130062821	-0,337074367	0,597200008
5 - 4	-0,301158488	-0,769516024	0,167199049	C 4	-0,21444994	-0,632085582	0,203185701
6 - 5	-0,166212873	-0,652437403	0,320011657	C 5	-0,328899035	-0,737690761	0,079892692
7 - 6	0,082069928	-0,547517106	0,711656962	C 6	-0,187875506	-0,716578249	0,340827237
8 - 7	-0,071607688	-0,72907496	0,585859584	C 7	-0,24037721	-0,793492865	0,312738445
9 - 8	0,037967637	-0,588921655	0,66485693	C 8	-0,183630103	-0,650080399	0,282820193
10 - 9	0,076839548	-0,48368185	0,637360945	C 9	-0,087528657	-0,543216019	0,368158706
11 - 10	0,336742281	-0,1999161	0,873400661	C 10	0,257523307	-0,159347962	0,674394575
12 - 11	-0,234627543	-0,781761602	0,312506517	C 11	-0,003441847	-0,456326996	0,449443302
13 - 12	0,77720624	0,268108344	1,286304137	C 12	0,774018003	0,438053717	1,10998229
Trkb							
**contrast.seq**	**estimate.seq**	**conf.low.seq**	**conf.high.seq**	**contrast.md**	**estimate.md**	**conf.low.md**	**conf.high.md**
2 - 1	0,788005777	-0,392502484	1,968514039	C 1	0,788005777	-0,387692931	1,963704485
3 - 2	-0,866510327	-2,621993506	0,888972853	C 2	-0,528793565	-2,202942834	1,145355704
4 - 3	-0,550620549	-1,585957257	0,484716159	C 3	-0,894951242	-2,089969485	0,300067001
5 - 4	-0,021835142	-0,965056331	0,921386046	C 4	-0,61387981	-1,582140407	0,354380786
6 - 5	0,925936816	-0,053394497	1,905268129	C 5	0,46195789	-0,434457311	1,358373091
7 - 6	0,983203269	-0,624388002	2,590794541	C 6	1,361568779	0,03726465	2,685872908
8 - 7	-0,057743351	-1,381944961	1,26645826	C 7	1,164177349	-0,152695857	2,481050554
9 - 8	0,373501555	-0,889131155	1,636134266	C 8	1,446011396	0,304211483	2,587811309
10 - 9	0,712418133	-0,416363665	1,841199931	C 9	2,005681846	0,921098028	3,090265665
11 - 10	1,131274388	0,050548096	2,212000681	C 10	2,945330581	1,95653677	3,934124392
12 - 11	-0,793886402	-1,895824901	0,308052098	C 11	1,848495891	0,825484541	2,87150724
13 - 12	-0,227651368	-1,264794202	0,809491466	C 12	1,483918901	0,705442769	2,262395033
Vegfa							
**contrast.seq**	**estimate.seq**	**conf.low.seq**	**conf.high.seq**	**contrast.md**	**estimate.md**	**conf.low.md**	**conf.high.md**
2 - 1	-0,165925237	-0,553402151	0,221551677	C 1	-0,165925237	-0,539463461	0,207612986
3 - 2	0,285939845	-0,575109408	1,146989097	C 2	0,217281126	-0,598275733	1,032837985
4 - 3	0,202672595	-0,141529377	0,546874566	C 3	0,345880609	-0,213325586	0,905086805
5 - 4	0,883850644	0,570275766	1,197425522	C 4	1,114437717	0,701041786	1,527833647
6 - 5	0,921155891	0,595561176	1,246750606	C 5	1,7665914	1,411849785	2,121333016
7 - 6	0,800721949	-0,00760084	1,609044737	C 6	2,250660173	1,63983085	2,861489496
8 - 7	0,315636286	-0,124605026	0,755877599	C 7	2,337415764	1,759330582	2,915500946
9 - 8	-0,221345689	-0,64111942	0,198428041	C 8	1,933459468	1,417181683	2,449737253

10 - 9	-0,020521233	-0,395780378	0,354737912	C 9	1,710127801	1,233383522	2,186872081
11 - 10	0,40331341	0,044030132	0,762596688	C 10	1,951087306	1,517847464	2,384327148
12 - 11	0,396670706	0,030325944	0,763015469	C 11	2,148214992	1,726153371	2,570276613
13 - 12	-0,129778228	-0,473421991	0,213865535	C 12	1,860147238	1,519259632	2,201034845
