# Supplementary material for: Adaptation of the Oxygen Sensing System during Lung Development
Source: Oxid Med Cell Longev. 2022 Feb 18;2022:9714669. doi: 10.1155/2022/9714669 (PMC8886745; doi:10.1155/2022/9714669)
Supplement: Supplementary 3 — Supplementary Figure S1: Supplementary Figure S2: effects of systemic hypoxia on HRG expression in rats. [file 9714669.f3.pdf]

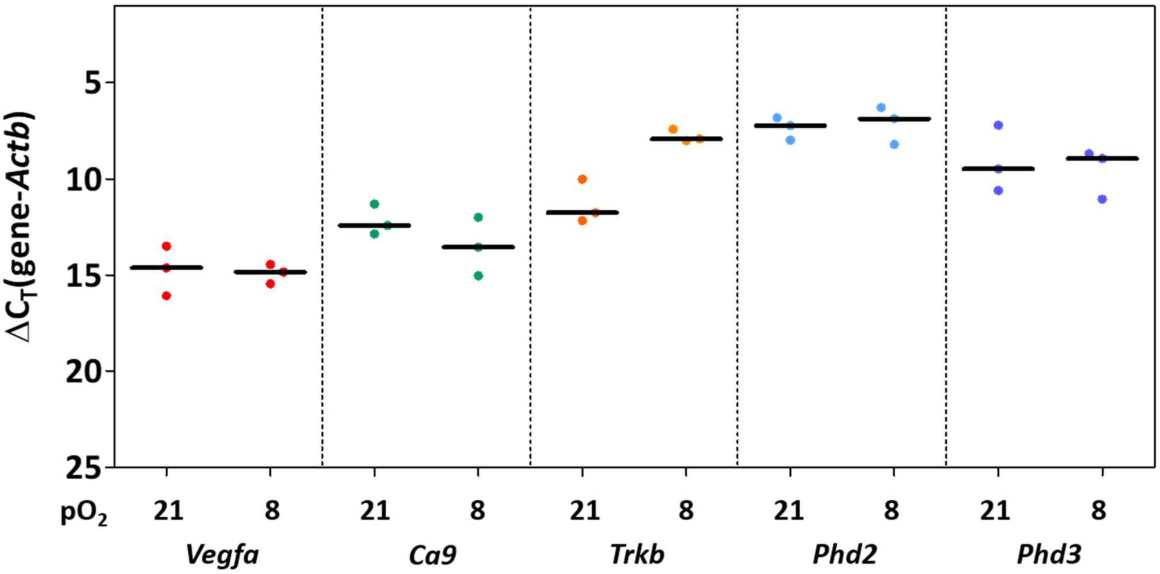

Supplementary Figure S1: **Effects of systemic hypoxia on HRG expression in rats.** Relative expression quantification of *bona fide* HRGs in adult rats exposed to 21% vs. 8% O_2 for 6 h, n=3 per condition. Statistical significance (Mann-Whitney-U test) in comparison to the respective controls is indicated by *p<0.05, **p<0.01, ***p<0.001. The black bar represents the median.
